# Associations of 24-h movement behavior with mental health in adolescent athletes: a compositional isotemporal substitution analysis

**DOI:** 10.3389/fpubh.2026.1852839

**Published:** 2026-05-29

**Authors:** Yunfeng Song, Ming Liu, Chi Xu

**Affiliations:** 1 Hubei Sports Science Research Institute (State Key Laboratory), Wuhan, Hubei, China; 2 School of Sports Health, Key Laboratory of Sports and Health Integration, Tianjin University of Sport, Tianjin, China

**Keywords:** 24-h movement behaviors, adolescent athletes, compositional data analysis, isotemporal substitution model, mental health

## Abstract

**Background:**

Adolescent athletes are exposed to high training loads and competitive pressure, which may increase the risk of psychological problems such as sport-related cognitive trait anxiety, mental fatigue, and other psychological symptoms. Understanding how 24-h movement behaviors—including moderate-to-vigorous physical activity (MVPA), light-intensity physical activity (LPA), sedentary behavior (SB), and sleep (SLP)—are associated with psychological outcomes is important for developing effective strategies to promote mental health in this population.

**Objective:**

This study aimed to examine the associations between 24-h movement behavior and sport-related cognitive trait anxiety, mental fatigue, and psychological symptoms among adolescent athletes, and to estimate model-based associations of time reallocation among these behaviors using a compositional isotemporal substitution approach.

**Materials and methods:**

A cross-sectional study was conducted among 136 adolescent athletes aged 12–17 years (69 males and 67 females) recruited from Wuhan Water Sports School in China. Twenty-four-hour movement behaviors, including MVPA, LPA, SB, and SLP, were objectively measured using triaxial accelerometers. Sport-related cognitive trait anxiety, mental fatigue, and psychological symptoms were assessed using the Cognitive Trait Anxiety Inventory for Athletes (CCTAI), the Athlete Mental Fatigue Scale, and the Athlete Psychological Symptoms Scale, respectively. Isotemporal substitution models were applied to examine the associations between time reallocation among 24-h movement behaviors and psychological outcomes.

**Results:**

SLP was negatively associated with sport-related cognitive trait anxiety, mental fatigue, and psychological symptoms (*β* = −2.357, *p* = 0.020; *β* = −2.349, *p* = 0.021; *β* = −2.265, *p* = 0.027). SB showed positive associations with sport-related cognitive trait anxiety, mental fatigue, and psychological symptoms (*β* = 1.863, *p* = 0.034; *β* = 1.722, *p* = 0.040; *β* = 1.663, *p* = 0.047). MVPA was negatively associated with cognitive trait anxiety and psychological symptoms (*β* = −1.831, *p* = 0.037; *β* = −1.662, *p* = 0.049), but positively associated with mental fatigue (*β* = 1.674, *p* = 0.045). In addition, LPA was negatively associated with mental fatigue (*β* = −1.669, *p* = 0.043).

**Conclusion:**

24-h movement behaviors were closely associated with psychological health among adolescent athletes. Reallocating time from SB to SLP may be associated with better psychological well-being. Schools, coaches, and parents should pay greater attention to the comprehensive influence of daily movement behavior patterns on the mental health of adolescent athletes.

## 1 Background

Adolescent athletes are at a critical stage of physical and psychological development and are exposed to dual career pressures arising from high-intensity sport-specific training and academic demands (1). In recent years, psychological problems such as sport-related cognitive trait anxiety, mental fatigue, and other psychological symptoms have become increasingly common in this population (2, 3). These challenges not only reduce athletes’ subjective well-being but may also impair athletic performance and long-term career development. Therefore, identifying strategies that help adolescent athletes maintain psychological adjustment under high-pressure conditions is important for promoting their healthy development.

Previous studies have primarily focused on the effects of training load or single psychological interventions in athletes, whereas the potential role of daily movement behaviors has received relatively limited attention (4). Daily movement behaviors, also referred to as 24-h movement behaviors (5), include moderate-to-vigorous physical activity (MVPA), light-intensity physical activity (LPA), sedentary behavior (SB), and sleep duration (SLP). The substitution patterns among these behaviors are considered important determinants of mental health outcomes (6, 7). The isotemporal substitution model (ISM) is a statistical approach used to estimate the independent effects of reallocating a fixed duration of time from one behavior to another while keeping the total time constant (8). However, this model traditionally treats each behavior as an independent variable and ignores the inherent interdependence among movement behaviors within a 24-h cycle, which may lead to multicollinearity in regression analyses (9). To address this limitation, compositional data analysis (CoDA) was introduced by Chastin et al. (10), providing a methodological framework that appropriately accounts for the compositional nature of time-use data and reduces multicollinearity problems.

Previous studies have applied compositional isotemporal substitution models to examine the effects of behavioral reallocation on mental health outcomes. Meyer et al. reported that reallocating time from SB to LPA or SLP was associated with psychological benefits among American adolescents (11). Kandola et al. found that replacing SB with MVPA significantly improved symptoms of depression and anxiety in adults (12). Similarly, Wang et al. demonstrated that substituting SB with MVPA during a 10-min class break positively influenced the mental health of junior high school students (13). These findings provide a theoretical basis for exploring the dose–response relationship between changes in physical behaviors and mental health among adolescent athletes.

Therefore, the present study focuses on the physical and psychological health development of adolescent athletes. A compositional isotemporal substitution model was applied to examine the association between the composition of 24-h movement behaviors and mental health outcomes, as well as the potential substitution benefits between different behaviors. We hypothesized that, under a constant 24-h time budget, reallocating time from SB to SLP or MVPA would significantly reduce sport-related cognitive trait anxiety, mental fatigue, and psychological symptoms in adolescent athletes.

## 2 Methods and materials

### 2.1 Study design and participants

Participants were recruited from Wuhan Water Sports School between December 2025 and February 2026. The participants were adolescent competitive athletes aged 12–17 years and were registered members of school-based sport-specific training teams. All participants received systematic sport-specific training in rowing or canoeing/kayaking and regularly participated in organized municipal-level competitions in Wuhan. They were all certified as National Level II athletes in China and had received specialized training for at least 1 year before enrollment. Their mean duration of specialized training was 3.82 ± 1.54 years, ranging from approximately 14 to 78 months. The participants’ athletic background, competition experience, training history, and qualification status were confirmed by the school and coaching staff.

A total of 145 adolescent athletes were initially enrolled in the study. An *a priori* sample size calculation was performed using G^*^Power 3.1.9.7. Because the primary analyses were multiple linear regression models based on isometric log-ratio coordinates, we selected F tests and “linear multiple regression: fixed model, R^2^ deviation from zero.” Assuming a medium effect size of f^2^ = 0.20, *α* = 0.05, and statistical power = 0.90, the required minimum sample size was 108. The final valid sample size of 136 exceeded this requirement. The inclusion criteria were as follows: (1) aged between 12 and 17 years; (2) currently receiving systematic sport-specific training in rowing or canoeing/kayaking at Wuhan Water Sports School; (3) having at least 1 year of specialized training experience; (4) being certified as a national second-level athlete in China; (5) regularly participating in organized municipal-level competitions in Wuhan; (6) being in good physical condition and able to complete all required tests; and (7) written informed consent obtained from the participants, their parents or legal guardians, and the school authorities. Participants were excluded if they were unable to participate in regular physical activity within 1 week prior to the assessment because of acute injury, illness, or other medical conditions.

After data screening, nine participants were excluded because their questionnaire responses failed to meet the quality-control criteria and were therefore considered invalid. Finally, 136 participants with valid accelerometer and questionnaire data were included in the final analysis, and the sample size was considered sufficient for the statistical analyses conducted in this study. The study protocol was approved by the Ethics Committee of the Hubei Institute of Sports Science (Approval No. 2025004). All methods were performed in accordance with the Declaration of Helsinki and the requirements of the institutional ethical review board.

### 2.2 24-h movement behaviors assessment

24-h movement behaviors were assessed using a triaxial accelerometer (ActiGraph GT3X+, ActiGraph LLC, Pensacola, FL, United States). Participants were instructed to wear the accelerometer on the right side of the waist for seven consecutive days and to remove it only during bathing, showering, swimming, or other situations in which the device could not be worn safely. Physical activity intensity was classified using the validated Evenson cut-off points for children and adolescents (14): sedentary behavior (SB), ≤100 counts/min; light-intensity physical activity (LPA), 101–2,295 counts/min; and moderate-to-vigorous physical activity (MVPA), ≥2,296 counts/min.

SLP was primarily estimated using accelerometer-derived sleep–wake data (15). Parent-reported daily sleep records were used as supplementary information to verify sleep timing and assist in the identification of potential non-wear periods. Given that the participants were adolescent athletes aged 12–17 years, their parents or legal guardians recorded bedtime, wake-up time, and any periods during which the accelerometer was removed on each monitoring day. These records were cross-checked against accelerometer-derived sleep–wake patterns to improve the accuracy of sleep-interval identification. The average daily sleep duration was calculated from the final verified weekday and weekend sleep-duration estimates using Equation 1 as follows:

$$\mathrm{SL}P_{\mathrm{avg}}=\frac{5\times \mathrm{SL}P_{\text{weekday}}+2\times \mathrm{SL}P_{\text{weekend}}}{7}$$ (1)

where SLP_avg_ represents the average daily sleep duration, SLP_weekday_ represents the mean daily sleep duration on weekdays, and SLP_weekend_ represents the mean daily sleep duration on weekend days.

### 2.3 Mental health assessment

Sport-related cognitive trait anxiety was assessed using the Competitive Cognitive Trait Anxiety Inventory (CCTAI) (16). The Chinese version of the CCTAI includes six sport-specific cognitive anxiety dimensions: social evaluation, competition preparation, performance concerns, failure consequences, opponent ability, and injury concerns. Previous Chinese standardization research reported that the CCTAI could distinguish sport-related cognitive trait anxiety across sex, sport type, and athletic level, supporting its known-group validity and applicability in Chinese athlete samples (17). In addition, a study of Chinese weightlifters further reported subscale-level internal consistency evidence for the CCTAI, with Cronbach’s *α* coefficients for the six dimensions ranging from 0.629 to 0.869, providing additional reliability evidence in a Chinese athlete sample (18). In the present study, the Cronbach’s α coefficient for the total CCTAI score was 0.86.

Mental fatigue was measured using the Athlete Burnout Questionnaire (ABQ) (19). The ABQ includes 15 items covering three dimensions: emotional/physical exhaustion, reduced sense of athletic accomplishment, and sport devaluation. Previous validation research on the Chinese translated ABQ included 214 Chinese collegiate athletes and 505 Chinese elite athletes, supported its multidimensional structure, and reported satisfactory reliability, with subscale Cronbach’s *α* coefficients above 0.70 and McDonald’s *ω* coefficients ranging from 0.856 to 0.927 (20). In addition, recent evidence from Chinese adolescent athlete samples has further supported the applicability of the ABQ in youth sport populations. For example, a study of Chinese adolescent soccer players aged 12–18 years reported good structural validity and reliability for the ABQ, with satisfactory model fit indices and Cronbach’s *α* coefficients above 0.70 for the total scale and all three dimensions (21). In the present study, the Cronbach’s α coefficient for the total ABQ score was 0.89.

Psychological symptoms were assessed using the Symptom Checklist-90 (SCL-90) (22). The SCL-90 contains 90 items measuring nine symptom dimensions, including somatization, obsessive-compulsive symptoms, interpersonal sensitivity, depression, anxiety, hostility, phobic anxiety, paranoid ideation, and psychoticism. The SCL-90 has been widely used and psychometrically examined in Chinese athlete populations, including young athletes, and has demonstrated acceptable reliability and validity. A recent cross-temporal meta-analysis of mental health among Chinese athletes from 1995 to 2023 included 28 studies with 4,227 Chinese athletes, all of which used the SCL-90 to evaluate athletes’ mental health status, indicating its extensive application in Chinese athlete samples (23). In addition, a recent study of Chinese college bridge athletes reported good psychometric properties for the SCL-90, with a Cronbach’s *α* coefficient of 0.951 and a KMO value of 0.928, supporting good internal consistency reliability and structural validity in an athlete sample (24). Another recent study involving 328 Chinese young athletes also used the SCL-90 to assess psychological symptoms and reported that the scale demonstrated acceptable reliability and validity among young athletes in China (25). In the present study, the Cronbach’s *α* coefficient for the total SCL-90 score was 0.93.

### 2.4 Quality control

To ensure data accuracy and reliability, all instruments were calibrated before data collection, and investigators received standardized training to maintain procedural consistency. After collection, questionnaires were reviewed for completeness and validity, and responses with missing items, obvious response patterns, or predefined exclusion criteria were removed. Data were entered independently by two researchers using a double-entry procedure, and discrepancies were cross-checked and resolved before analysis.

### 2.5 Statistical analysis

All statistical analyses were performed using R software (version 4.5.2). The 24-h movement behaviors, including MVPA, LPA, SB, and SLP, were treated as compositional data because these behaviors are mutually exclusive and constrained to a fixed 24-h time budget. Thus, an increase in time spent in one behavior necessarily corresponds to a decrease in time spent in one or more of the remaining behaviors. CoDA was used to appropriately account for this co-dependent structure of daily time-use data (26).

First, the distribution characteristics of the 24-h movement behaviors were described using the geometric mean and the variation matrix of compositional data (27). The geometric mean was used to represent the central tendency of the compositional data, while the variation matrix was used to quantify the relative variability between movement behaviors.

Second, compositional regression models were constructed to examine the associations between the overall 24-h movement behavior composition and mental health outcomes. Because raw compositional parts are linearly dependent and cannot be entered directly into conventional regression models, the four-part composition was transformed from the simplex space to the Euclidean space using isometric log-ratio (ilr) transformation (9). This transformation generated three ilr coordinates, which were then entered into multiple linear regression models as predictors. Sport-related cognitive trait anxiety, mental fatigue, and psychological symptoms were used as dependent variables, and age and sex were included as covariates. In this framework, the regression coefficients represent associations between relative differences in the overall 24-h movement behavior composition and mental health outcomes, rather than the independent effect of any single isolated behavior.

Third, compositional isotemporal substitution analyses were conducted to improve the practical interpretability of the compositional regression models. While CoDA accounts for the relative and constrained nature of 24-h time-use data, isotemporal substitution translates the fitted compositional regression models into hypothetical time-reallocation scenarios. Specifically, new 24-h compositions were generated by increasing one behavior by 15 min and decreasing another behavior by the same amount, while holding the total time constant at 24 h. Each substituted composition was then transformed using the same ilr procedure and entered into the fitted regression model to estimate the predicted difference in psychological outcomes compared with the reference composition. Thus, the substitution estimates represent model-predicted changes associated with reallocating time between behaviors, rather than observed intervention effects. To further examine potential dose–response patterns, additional substitution scenarios were generated by reallocating time between behaviors in 5-min increments, ranging from −30 to 30 min. Predicted changes in mental health outcomes were then plotted to illustrate the direction and magnitude of associations across different reallocation doses. All statistical tests were two-sided, and statistical significance was set at *α* = 0.05.

## 3 Results

### 3.1 Distribution of 24 h movement

#### 3.1.1 Descriptive analysis

The arithmetic mean slightly overestimated the proportions of MVPA (6.88%) and LPA (13.83%), while slightly underestimating SB (45.51%) and SLP (33.78%). The compositional geometric mean indicated that the distribution of the movement behaviors conformed to the assumptions of compositional data, suggesting that the data were suitable for compositional data analysis (See Table 1).

**Table 1:** Compositional mean and arithmetic mean of 24-h movement data.

Statistic	Arithmetic mean (min/%)	Compositional mean (min/%)
MVPA	99.05(6.88)	92.15(6.40)
LPA	199.12(13.83)	197.44(13.71)
SB	655.33(45.51)	657.21(45.64)
SLP	486.50(33.78)	493.20(34.25)

#### 3.1.2 Variance matrix analysis

**Figure 1:**
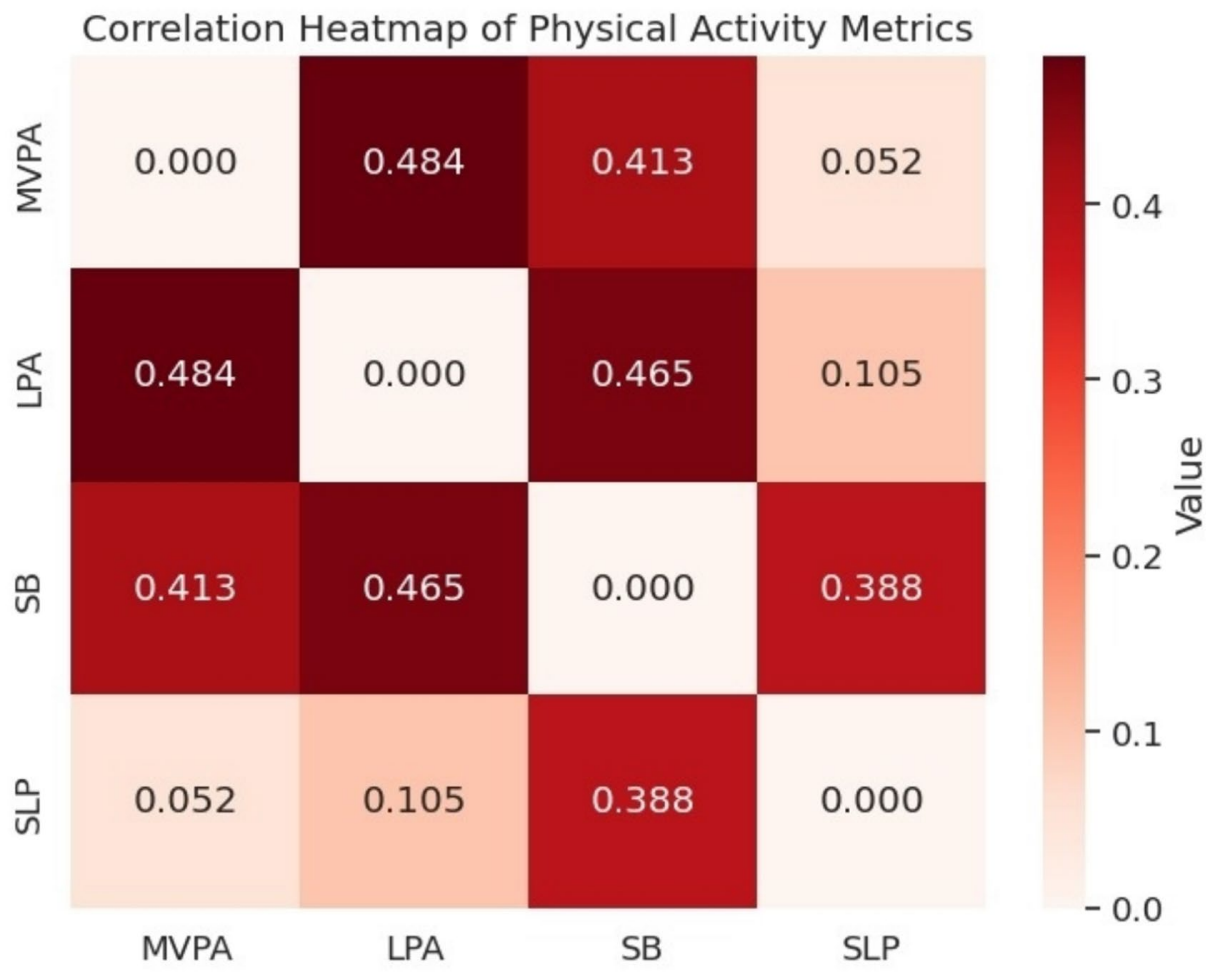
Variance matrix analysis.

The smallest log-ratio variance was found between MVPA and SLP (ln MVPA/SLP = 0.052), suggesting that these two behaviors tended to vary proportionally and were more likely to be reallocated relative to each other. In contrast, the largest log-ratio variance was observed between MVPA and LPA (ln MVPA/LPA = 0.484), indicating that these behaviors were less likely to substitute for one another (See Figure 1).

### 3.2 Compositional linear regression analysis of 24 h movement and mental health

**Figure 2:**
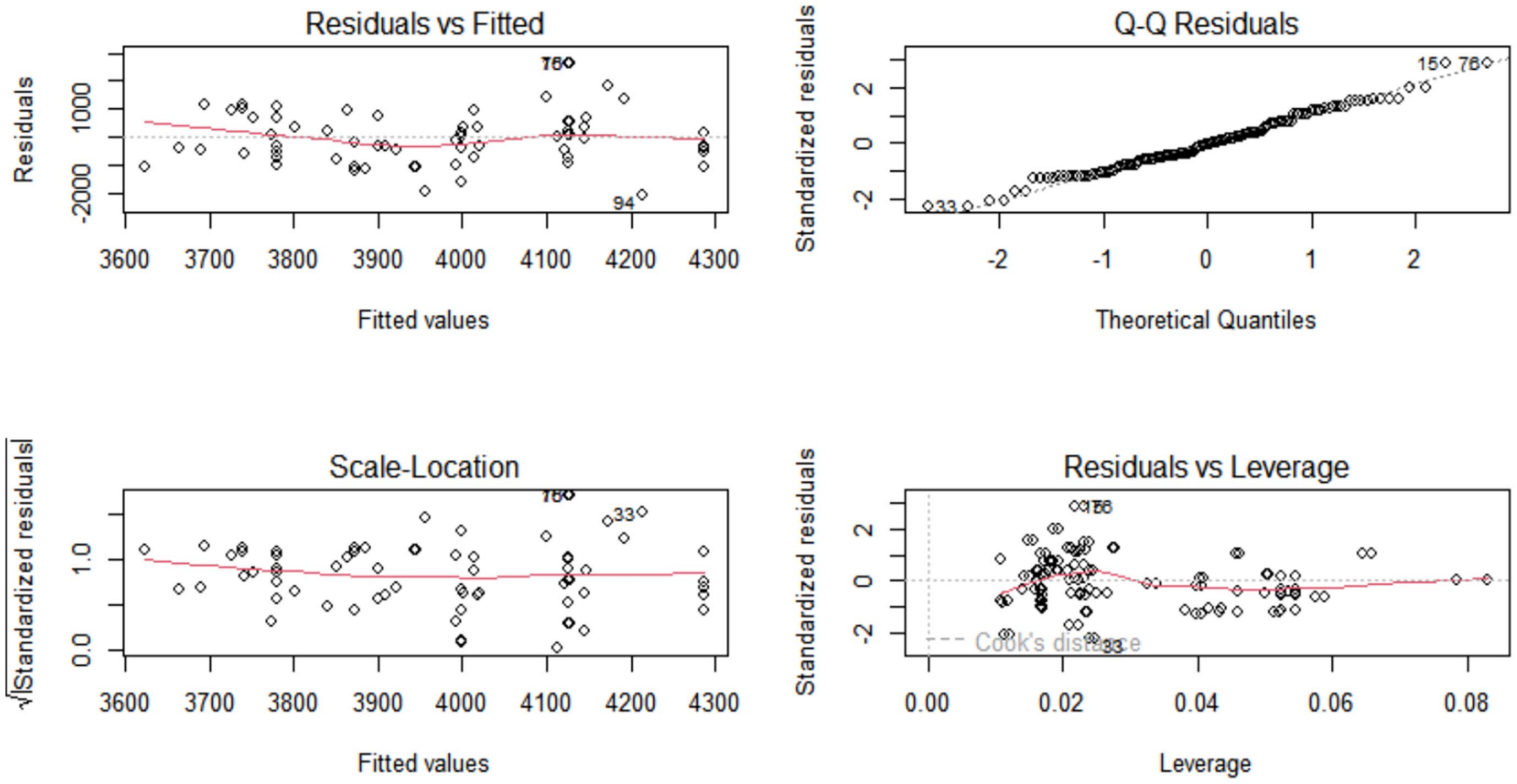
Linear regression diagnostics: residual analysis and influence point assessment.

Model diagnostics were assessed using residual plots, Q-Q plots, scale-location plots, and residuals versus leverage plots. The residuals were generally randomly distributed around zero, and no clear nonlinear pattern or severe heteroscedasticity was observed. The Q-Q plot indicated that the residuals were approximately normally distributed, although slight deviations were observed at the tails. No observations exceeded the Cook’s distance threshold, suggesting that no highly influential points were detected. Overall, the diagnostic plots indicated that the regression assumptions were reasonably satisfied (See Figure 2).

After adjusting for potential confounders including age and sex, compositional data analysis was conducted to examine the associations between 24-h movement behaviors and psychological outcomes. MVPA, LPA, SB, and SLP were included as independent variables, while sport-related cognitive trait anxiety, mental fatigue, and psychological symptoms were treated as dependent variables. In addition, a *post-hoc* statistical power analysis was performed using G^*^Power 3.1.9.7. Based on the final sample size of 136 participants, an alpha level of 0.05, an effect size of f^2^ = 0.20, and five predictors in the adjusted regression models, the achieved statistical power was approximately 98.8%, indicating that the sample size provided sufficient power to detect moderate associations in the primary analyses.

SLP was negatively associated with sport-related cognitive trait anxiety, mental fatigue, and psychological symptoms (*β* = −2.357, *p* = 0.020; *β* = −2.349, *p* = 0.021; *β* = −2.265, *p* = 0.027). SB showed positive associations with sport-related cognitive trait anxiety, mental fatigue, and psychological symptoms (*β* = 1.863, *p* = 0.034; *β* = 1.722, *p* = 0.040; *β* = 1.663, *p* = 0.047). MVPA was negatively associated with cognitive trait anxiety and psychological symptoms (*β* = −1.831, *p* = 0.037; *β* = −1.662, *p* = 0.049), but positively associated with mental fatigue (*β* = 1.674, *p* = 0.045). In addition, LPA was negatively associated with mental fatigue (*β* = −1.669, *p* = 0.043; See Table 2).

**Table 2:** Compositional linear regression between the proportion of 24-h movement and mental health outcomes.

Outcome	MVPA		LPA		SB		SLP		Overall model *p*
	*β_1_*	*p*	*β_2_*	*p*	*β_3_*	*p*	*β_4_*	*p*	
Sport-related cognitive trait anxiety	−1.831	0.037	−0.723	0.486	−2.357	0.020	1.863	0.034	<0.01
Mental fatigue	1.674	0.045	−1.669	0.043	−2.349	0.021	1.722	0.040	
Psychological symptoms	−1.662	0.049	−1.173	0.268	−2.265	0.027	1.663	0.047	

The model adjusted for covariates including age and sex. β represents the association between a change in a given behavior (relative to the other behaviors) and the mental health outcome. For example, β₁ represents the association between a change in MVPA (relative to SLP, SB, and LPA) and the mental health outcome.

### 3.3 Isotemporal substitution of 24-h movement behaviors and psychological outcomes

Regarding the effects of time reallocation on sport-related cognitive trait anxiety, mental fatigue, and psychological symptoms, the isotemporal substitution analysis revealed several significant associations. Reallocating time from SLP to MVPA, SB, or LPA was associated with significant increases in sport-related cognitive trait anxiety (*β* = 6.389, 7.148, and 6.261, respectively; all *p* < 0.05), mental fatigue (*β* = 2.426, 2.642, and 2.469, respectively; all *p* < 0.05), and psychological symptoms (*β* = 5.491, 6.785, and 5.756, respectively; all *p* < 0.05).

Conversely, increasing SLP by reallocating time from MVPA, SB, or LPA was associated with significant reductions in sport-related cognitive trait anxiety (*β* = −7.443, −8.268, and −7.353, respectively; all *p* < 0.05), mental fatigue (*β* = −3.349, −3.721, and −3.309, respectively; all *p* < 0.05), and psychological symptoms (*β* = −7.409 and −8.490, respectively; both *p* < 0.05). Among these substitution scenarios, reallocating time from SB to SLP showed the strongest beneficial effect.

In addition, replacing MVPA or SB with LPA, as well as replacing SB with MVPA, was associated with significant reductions in mental fatigue (*β* = −1.185, −0.990, and −1.670, respectively; all *p* < 0.05). Conversely, reallocating time in the opposite direction resulted in significant increases in mental fatigue (*β* = 1.365, 1.392, and 1.375, respectively; all *p* < 0.05). Furthermore, replacing SB with MVPA was associated with a reduction in psychological symptoms (*β* = −4.015, *p* < 0.05), whereas reallocating time from MVPA to SB produced the opposite effect (*β* = 4.015, *p* < 0.05; See Table 3).

**Table 3:** Predicted changes in psychological outcomes after a 15-min isotemporal substitution among 24-h movement behaviors (95%CI).

Outcome		SLP↑	MVPA↑	SB↑	LPA↑
		*β*(95%CI)	*β*(95%CI)	*β*(95%CI)	*β*(95%CI)
Sport-related cognitive trait anxiety	SLP↓		6.389(4.262–8.516)^*^	7.148(3.424–10.872)^*^	6.261(3.914–8.608)^*^
	MVPA↓	−7.443(−11.042–−3.843)^*^		1.825(−1.071–4.721)	2.712(−1.208–4.217)
	SB↓	−8.268(−12.209–−4.327)^*^	−1.925(−6.835–2.985)		−1.835(−3.4870–0.182)
	LPA↓	−7.353(−10.652–−4.054)^*^	−3.087(−4.632–1.543)	2.215(−0.641–3.789)	
Mental fatigue	SLP↓		2.426(1.834–3.018)^*^	2.642(1.151–4.133)^*^	2.469(1.117–3.821)^*^
	MVPA↓	−3.349(−4.969–−1.729)^*^		1.375(1.071–3.821)^*^	−1.185(−2.338–−0.030)^*^
	SB↓	−3.721(−5.494–−1.947)^*^	−1.670(−2.565–−0.775)^*^		−0.990(−1.945–−0.035)^*^
	LPA↓	−3.309(−4.793–−1.824)	1.365(0.121–2.611)^*^	1.392(1.145–1.639)^*^	
Psychological symptoms	SLP↓		5.491(3.456–7.526)^*^	6.785(4.040–9.530)^*^	5.756(3.602–7.910)^*^
	MVPA↓	−7.409(−11.593–−3.226)^*^		4.015(1.375–6.655)^*^	1.042(−1.288–3.371)
	SB↓	−8.49(−13.069–−3.829)^*^	−5.402(−8.759–−2.045)^*^		0.892(−1.667–3.451)
	LPA↓	−7.296(−11.101–−3.492)^*^	−1.780(−4.173–0.610)	−1.515(−3.954–0.923)	

The reported estimates represent predicted changes in the original psychological outcome scores under a 15-min time reallocation scenario, as derived from the fitted compositional regression model.*indicates statistical significance at *P* < 0.05.

### 3.4 Dose–response relationship of isotemporal substitution between SLP and other 24-h movement behaviors

**Figure 3:**
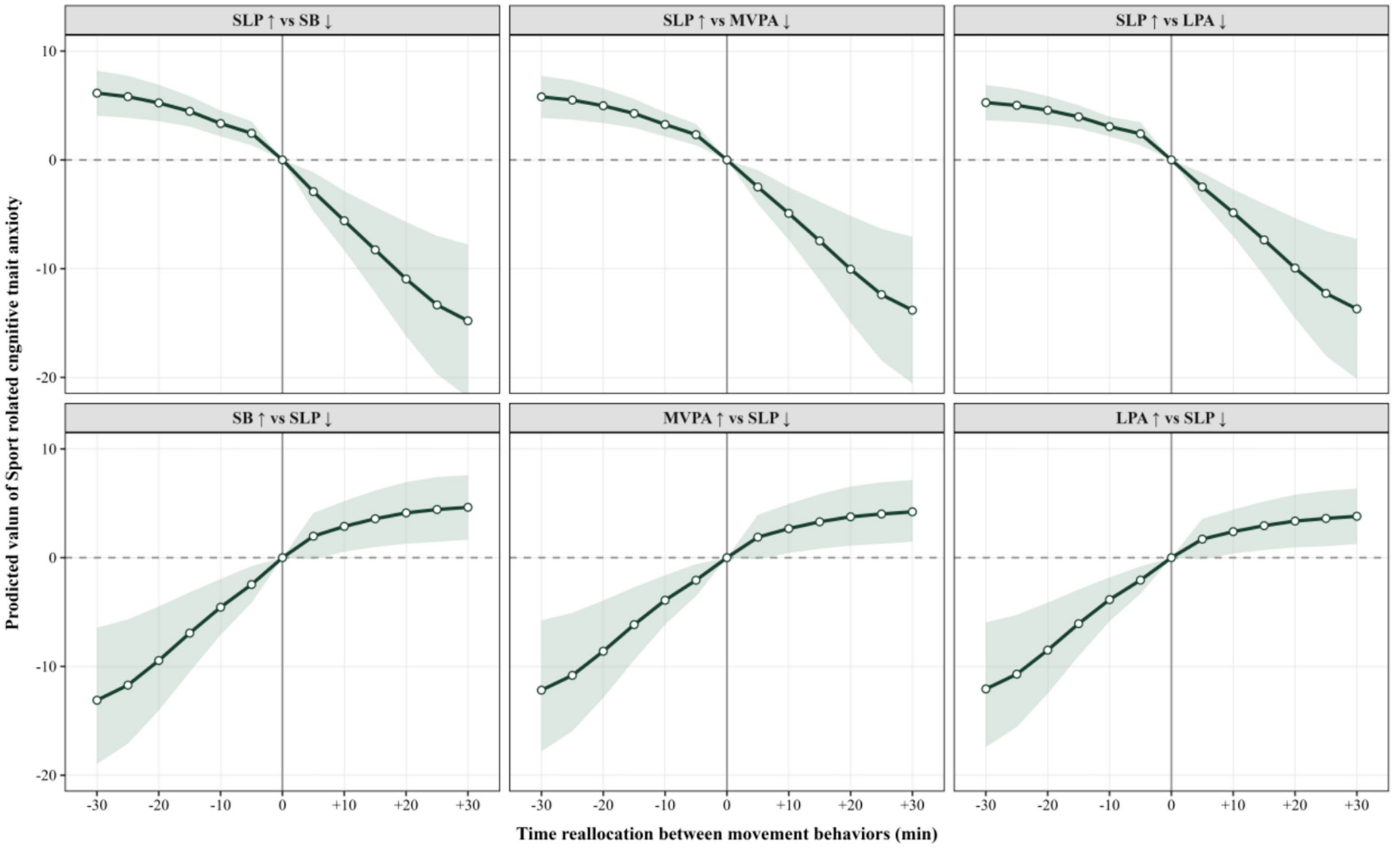
Reallocating SLP and other 24-h movement behaviors with sport-related cognitive trait anxiety.

**Figure 4:**
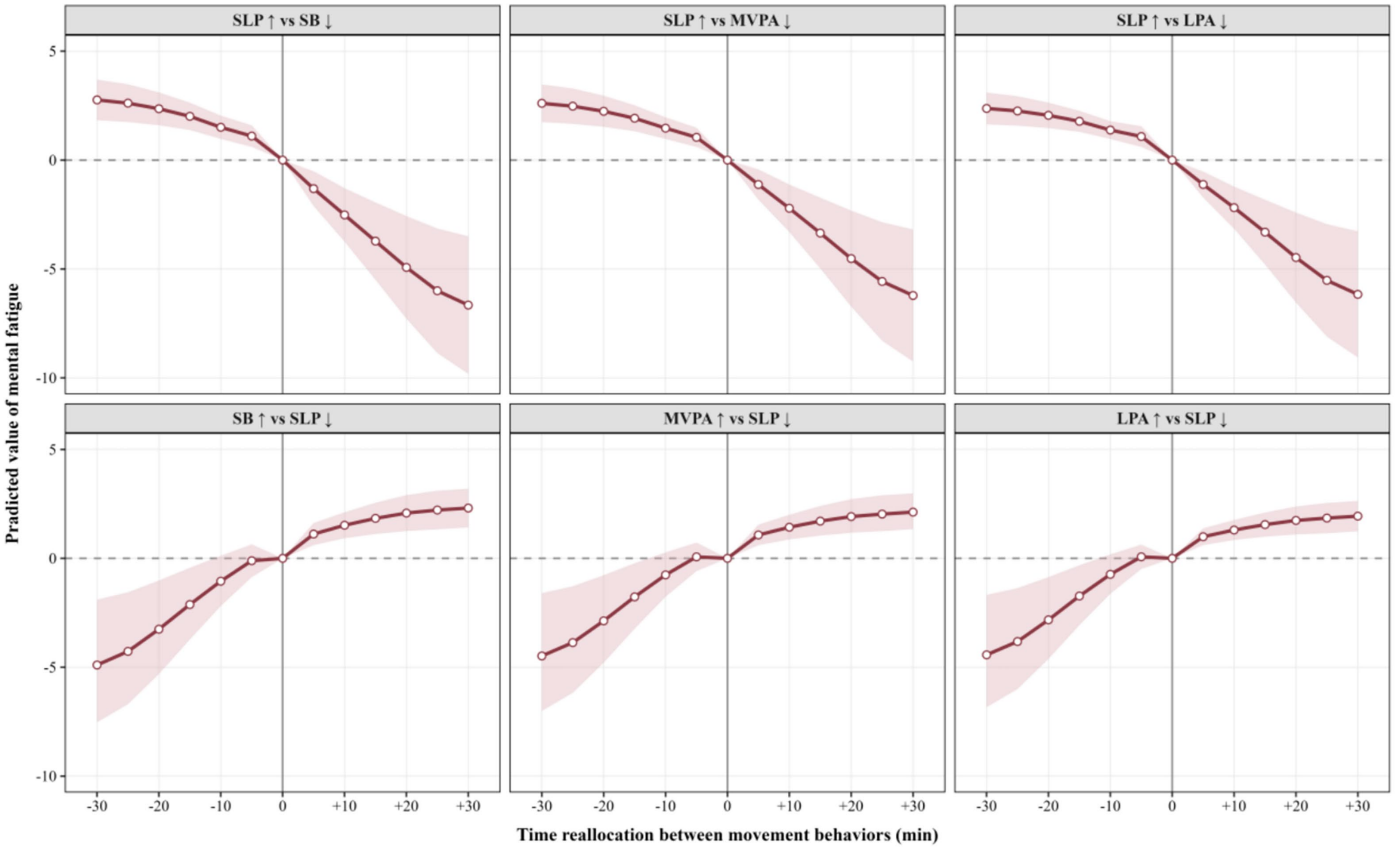
Reallocating SLP and other 24-h movement behaviors with mental fatigue.

**Figure 5:**
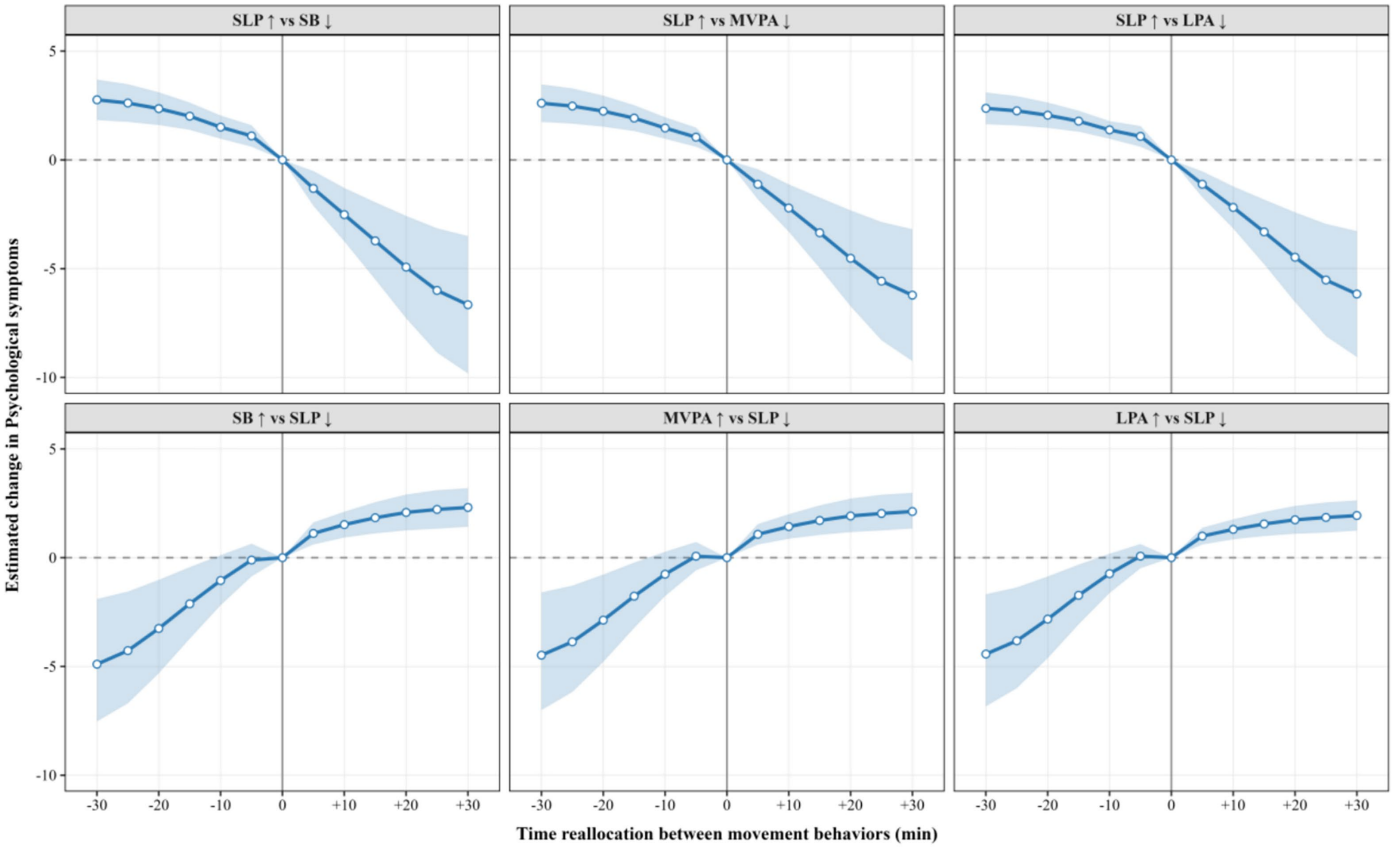
Reallocating SLP and other 24-h movement behaviors with psychological symptoms.

To further examine dose–response patterns, trend plots were generated to show predicted changes in psychological outcomes when time was reallocated between behavior pairs in 5-min increments from −30 to 30 min (Figures 3–5). Two main patterns emerged. First, substitutions involving SLP were asymmetric: reallocating time from MVPA, SB, or LPA to SLP was associated with greater improvements in psychological outcomes than the corresponding reverse substitutions. Second, the beneficial associations became more pronounced when SLP replaced at least 25 min/day of other behaviors. Improvements increased rapidly during the 5–25 min range but tended to plateau between 25 and 30 min, suggesting a diminishing marginal effect.

## 4 Discussion

This study addressed the limitations of traditional approaches that analyze movement behaviors in isolation by applying a compositional isotemporal substitution model to examine the associations and substitutions between 24-h movement behaviors and psychological health among adolescent athletes.

The participants in this study spent an average of 92.15, 197.44, 657.21, and 493.20 min/day in MVPA, LPA, SB, and SLP, respectively. Compared with previous studies, adolescent athletes showed longer MVPA and SB but shorter LPA and SLP durations. This pattern challenges the assumption that high physical activity necessarily corresponds to low sedentary behavior. Previous studies have similarly shown that individuals may meet MVPA recommendations while still spending much of their waking time sedentary (28). Although athletes engage in structured high-intensity training, they may accumulate substantial sedentary time during non-training periods due to academic demands, recovery needs, or reduced motivation for additional light activity. In particular, athletes’ sedentary behavior has been reported to occur mainly in work, reading, or study contexts during weekdays, whereas mobile phone use and television viewing are major contributors during non-working days (29). Therefore, both training-related recovery periods and educational settings may represent important contexts for sedentary accumulation among adolescent athletes, suggesting that future interventions should target unnecessary sedentary time without compromising training recovery.

The variation matrix further provided useful context for interpreting potential reallocations among 24-h movement behaviors (30). Sedentary behavior appeared relatively stable among adolescent athletes, suggesting limited flexibility for substitution within the 24-h time budget, possibly due to their highly structured daily routines. Notably, the low log-ratio variance between MVPA and SLP indicates a stable proportional relationship between these behaviors, suggesting that they may co-vary within a balanced behavioral structure rather than change independently. Practically, high-intensity MVPA may increase physiological fatigue and sleep need, whereas limited time resources may lead athletes to sacrifice sleep for additional training opportunities (31). Thus, substitution patterns involving MVPA and SLP should be interpreted within this dynamic but proportionally stable 24-h behavioral balance. These findings strengthen the compositional interpretation of the subsequent dose–response substitution analyses.

Using compositional data analysis with isometric log-ratio transformation, this study found that SLP was negatively associated with sport-related cognitive trait anxiety, mental fatigue, and psychological symptoms, whereas SB was positively associated with these outcomes, consistent with previous studies (32, 33). These findings suggest that increasing sleep and reducing sedentary time may benefit psychological health among adolescent athletes. Isotemporal substitution analysis further showed that replacing SB with MVPA was associated with fewer psychological symptoms, supporting evidence that positive exercise behaviors may promote mental health (34). However, replacing LPA with MVPA was associated with higher mental fatigue, differing from findings in general adolescents, for whom MVPA often occurs during school breaks or physical education classes and may help regulate emotions and relieve stress (35). Among adolescent athletes, however, MVPA more likely reflects structured, high-intensity training. Thus, its positive association with mental fatigue should be interpreted cautiously and may reflect higher training intensity, accumulated training load, or insufficient recovery rather than an adverse effect of MVPA itself (36). These findings suggest that mental health promotion in adolescent athletes should emphasize the balance between training, recovery, sleep, and sedentary time, rather than simply increasing MVPA.

Furthermore, SLP was negatively associated with sport-related cognitive trait anxiety, mental fatigue, and psychological symptoms, suggesting that sleep may play a key protective role in the psychological health of adolescent athletes. Adequate sleep supports emotional regulation and stress recovery, which is particularly important for athletes exposed to both academic demands and competitive pressure (37). In contrast, insufficient sleep may impair stress-coping capacity and disrupt emotional regulation, thereby increasing vulnerability to anxiety and fatigue (38). Within the 24-h movement behavior framework, sleep should therefore be prioritized when optimizing behavioral composition. Interventions for adolescent athletes should move beyond simply increasing physical activity and instead promote an appropriate balance among training, recovery, sleep, and sedentary time. Coaches and parents should emphasize sleep hygiene and age-appropriate sleep duration, particularly after intensive training (39). Meanwhile, MVPA should be prescribed cautiously, as greater exercise volume does not necessarily lead to better psychological outcomes. Training programs should apply periodization principles and balance high-intensity training with sufficient recovery to reduce psychological risks associated with excessive training loads (40).

The dose–response analyses of reallocating time between SLP and other movement behaviors revealed two main findings. First, substitutions involving SLP showed clear bidirectional asymmetry, a pattern also reported in previous compositional studies (41, 42). Because isotemporal substitution estimates are derived from compositional models and expressed as predicted changes under hypothetical time-reallocation scenarios on the original minute scale, opposite substitutions should not be interpreted as simple mirror-image effects. Reallocating time within a fixed 24-h composition changes the relative balance among all behaviors; therefore, the observed asymmetry may partly reflect the statistical properties of the compositional substitution model. Nevertheless, the direction and magnitude of the estimates may also indicate meaningful behavioral patterns in adolescent athletes. The most relevant contrast was observed between SB and SLP: replacing SB with SLP was associated with decreases in sport-related cognitive trait anxiety, mental fatigue, and psychological symptoms, whereas replacing SLP with SB was associated with increases in these outcomes. This pattern suggests that increasing sleep at the expense of sedentary time may be particularly protective for psychological health (43). Given the sleep debt commonly observed in athletes due to training, competition, and academic demands, additional sleep may support psychological recovery, whereas short-term sleep reduction may be partly buffered by resilience and adaptive recovery strategies (18). Second, the beneficial association was most pronounced when at least 25 min/day of SB was replaced by SLP. Compared with traditional recommendations requiring larger behavioral changes, such as increasing SLP by 1 hper day, reallocating approximately 25 min from SB to SLP may be more feasible for adolescent athletes. Coaches and sports administrators may therefore consider delaying early training sessions, optimizing evening schedules, and reducing unnecessary sedentary activities, such as excessive screen use or inefficient meetings, to help athletes obtain modest but meaningful additional SLP (44).

## 5 Limitations

This study also has several limitations. First, given the exploratory nature of this study and the relatively small sample size, no formal adjustment for multiple comparisons was applied. Therefore, findings from multiple association and isotemporal substitution analyses should be interpreted cautiously, with emphasis placed on the direction, magnitude, and consistency of the observed associations rather than on isolated *p* values. In addition, the cross-sectional design only allows for the identification of associations rather than causal relationships. Second, the outcome variables were primarily assessed through self-reported questionnaires, which may introduce potential bias. Future studies may incorporate physiological indicators, such as heart rate variability, to provide more precise assessments of fatigue and recovery. Third, the models adjusted only for age and sex; individual training load, recovery status, sleep quality, and academic demands were not fully measured. Fourth, participants were recruited from a single water sports school, which may limit generalizability to athletes from other regions, sports, or training systems.

## 6 Conclusion

24-h movement behaviors were closely associated with psychological health among adolescent athletes. Reallocating time from SB to SLP may be associated with better psychological well-being. Schools, coaches, and parents should pay greater attention to the comprehensive influence of daily movement behavior patterns on the mental health of adolescent athletes.

## Data Availability

The raw data supporting the conclusions of this article will be made available by the authors, without undue reservation.
